# Supplementary material for: Preoperative Multiparametric Quantitative Magnetic Resonance Imaging Correlates with Prognosis and Recurrence Patterns in Pancreatic Ductal Adenocarcinoma
Source: Cancers (Basel). 2022 Aug 31;14(17):4243. doi: 10.3390/cancers14174243 (PMC9454581; doi:10.3390/cancers14174243)
Supplement: Supplementary file 1 [file cancers-14-04243-s001.zip › Figure S1-S3.pdf]

Figure S1-S3 Related immunohistochemical and correlation analysis results

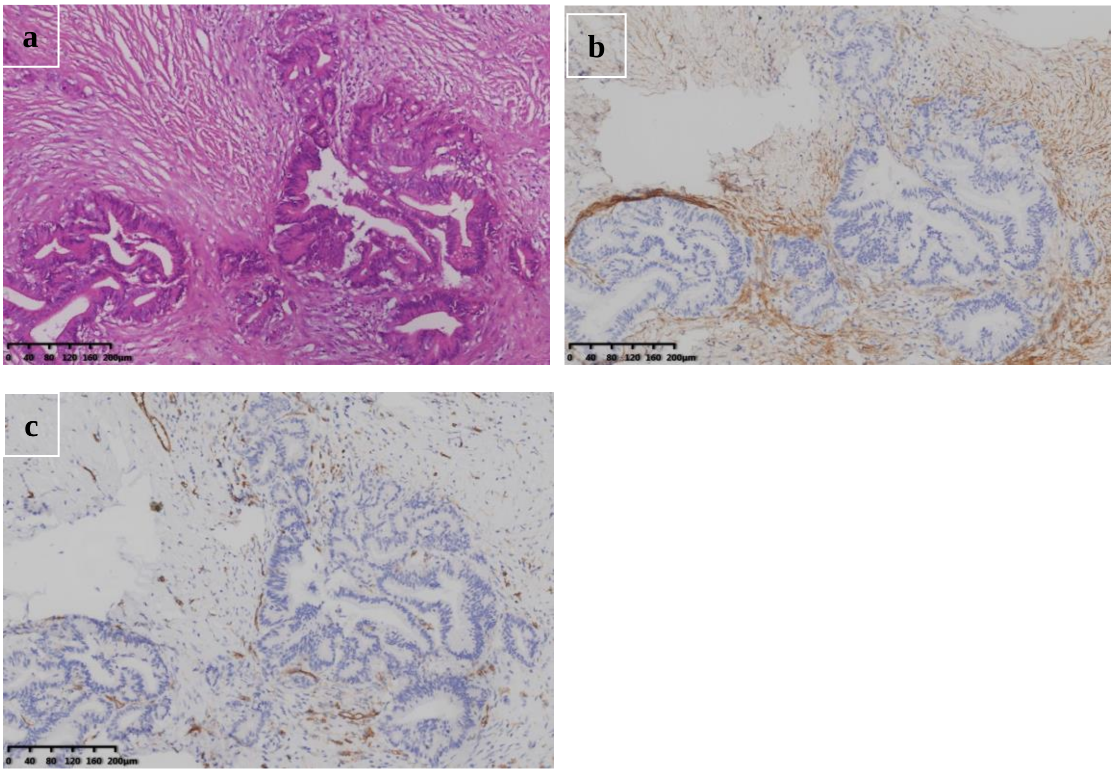

Figure S1. HE staining of one PDAC case (a,HE,x10). Immunohistochemical staining of anti-collage type I showed that the collagen content is not abundant(b,collagen,x10) and fewer capillaries(c,CD31,x10)in the tumor microenvironment.

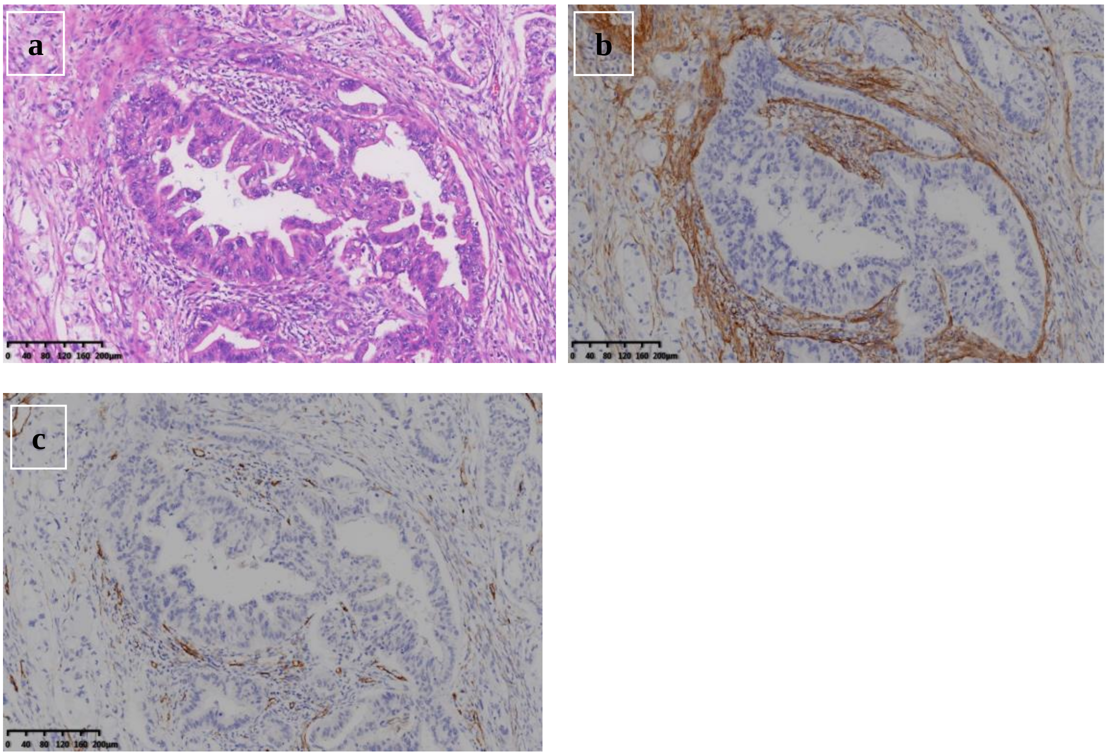

Figure S2.HE staining of one PDAC case. (a,HE,x10). Immunohistochemical staining showed that the collagen content was abundant (b,collagen,x10) and more capillaries (c,CD31,x10)in the tumor microenvironment.

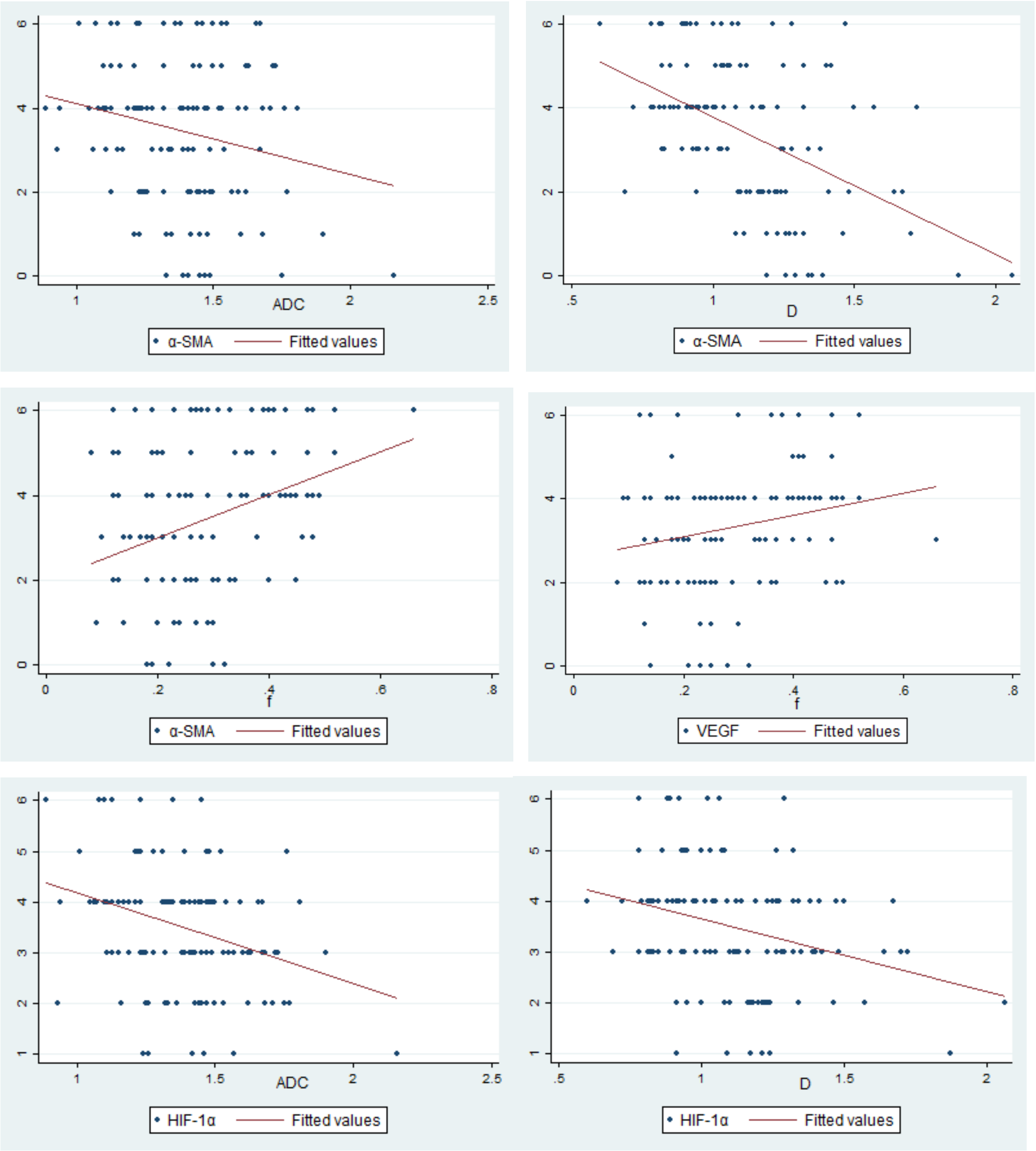

Figure S3. The Pearson correlation analysis between quantitative MRI parameters and the histopathology of PDAC.
