# Supplementary material for: Preoperative Multiparametric Quantitative Magnetic Resonance Imaging Correlates with Prognosis and Recurrence Patterns in Pancreatic Ductal Adenocarcinoma
Source: Cancers (Basel). 2022 Aug 31;14(17):4243. doi: 10.3390/cancers14174243 (PMC9454581; doi:10.3390/cancers14174243)
Supplement: Supplementary file 1 [file cancers-14-04243-s001.zip › Table S1.pdf]

Table S1. Cox Survival Analysis of Predictors of Recurrence-Free Survival.

Variables	Category	Univariate analysis		Multivariate analysis	
		HR (95% CI)	P value	HR (95% CI)	P value
Age(years)	＜65vs. ≥65	0.746(0.498,1.119)	0.157		
Sex	Male vs. Female	1.072(0.718,1.599)	0.734		
CEA(ng/ml)	≤5 vs.＞5	1.028(0.591,1.787)	0.923		
CA19-9(U/ml)	≤39 vs.＞39	0.793(0.438,1.248)	0.258		
After surgery CA19-9(U/ml)	≤39 vs.＞39	1.418(0.948,2.121)	0.089		
Type of surgery	PD	Ref			
	DP	1.241(0.799,1.925)	0.336		
	TP	1.953(0.931,4.094)	0.076		
Tumor location	Head	Ref			
	Body	0.919(0.397,2.124)	0.842		
	Tail	1.162(0.741,1.823)	0.513		
Tumor size(cm)	≤ 2	Ref		Ref	
	2-4	2.092(1.100,3.980)	**0.024**	1.993(1.040,3.818)	**0.038**
	＞4	3.117(1.491,6.515)	**0.003**	2.642(1.256,5.555)	**0.010**
N stage	N0	Ref			
	N1	1.465(0.963,2.228)	0.074		
	N2	1.742(0.878,3.457)	0.112		
AJCC stage, 8th,	IA	Ref			
	IB	2.045(0.905,4.623)	0.086		
	IIA	1.849(0.584,5.855)	0.296		
	IIB	2.602(1.165,5.809)	**0.020**		
	III	3.277(1.264,8.494)	**0.015**		
Baseline resectability	Resectable vs. Borderline	1.156(0.741,1.803)	0.522		
Margin	R0 vs. R1	1.767(1.162,2.685)	**0.008**	1.759(1.149,2.692)	**0.009**
Nerve invasion	(+) vs. (-)	0.928(0.516,1.666)	0.801		
Adjuvant Chemotherapy	Yes vs. No	0.761(0.507,1.143)	0.189		
ADC ($\times 10^{-3}$ mm ^{2}/s)	＞1.33 vs. ≤1.33	1.621(1.086,2.420)	**0.018**	1.238(0.781,1.964)	0.364
D($\times 10^{-3}$ mm ^{2}/s)	＞1.08 vs. ≤1.08	2.474(1.616,3.787)	**0.001**	2.066(1.252,3.409)	**0.005**
D*($\times 10^{-3}$ mm ^{2}/s)	＞7.94 vs. ≤7.94	1.008(0.649,1.565)	0.972		
f	＞0.28 vs. ≤0.28	0.598(0.399,0.895)	**0.012**	0.818(0.517,1.294)	0.391
DDC	＞1.5 vs. ≤1.5	1.390(0.903,2.140)	0.135		
α	＞0.86 vs. ≤0.86	1.441(0.872,2.382)	0.154		
